# Supplementary material for: Complexome profiling of the Chlamydomonas psb28 mutant reveals TEF5 as an early PSII assembly factor
Source: Plant Cell. 2025 Mar 17;37(6):koaf055. doi: 10.1093/plcell/koaf055 (PMC12164590; doi:10.1093/plcell/koaf055)
Supplement: koaf055_Supplementary_Data [file koaf055_supplementary_data.zip › psb28_interactive.zip!/plots/intensity/qualityPlots/peptides/GDH2_Cre05.g232150.t1.2.html]

### Cre05.g232150.t1.2

Trivial name: GDH2  
SeqCoverage [median]: 34.40  
Mass: 48.78 kDa  
  
 

| Sequence | MissedCleavage | Mass [Da] | wtIntensity | muIntensity | Score |
| --- | --- | --- | --- | --- | --- |
| EIIGTYEDIPAPDMNTDAK | 0 | 2091.97 | 1114.30 | 0.00 | 48.88 |
| EMNVPLR | 0 | 857.44 | 0.00 | 794.19 | 88.73 |
| GFSPGVVTGKPVYLHGSLGR | 1 | 2027.10 | 771.32 | 0.00 | 31.46 |
| GGVTVDPR | 0 | 799.42 | 2241.38 | 2815.09 | 111.82 |
| GTTFAIR | 0 | 764.42 | 4331.73 | 1033.53 | 100.67 |
| HVASGKPLAEFTGGAAVPK | 1 | 1835.99 | 1546.89 | 767.16 | 50.05 |
| KLVVAIK | 1 | 769.54 | 2134.13 | 0.00 | 84.36 |
| LDYPEK | 0 | 763.38 | 546.48 | 0.00 | 86.09 |
| LQNLLLTPR | 0 | 1066.65 | 1102.45 | 2334.81 | 158.87 |
| TAAFVVALQR | 0 | 1074.62 | 6468.56 | 6607.20 | 222.19 |
| TAVMDIPYGGAK | 0 | 1221.61 | 3952.24 | 3252.98 | 100.48 |
| YHPQVDLDDVR | 0 | 1355.65 | 4324.28 | 5972.39 | 110.84 |
|  |  |  | 28533.8 | 23577.3 |  |
